# Membrane-Based Hybrid Method for Purifying PEGylated Proteins

**DOI:** 10.3390/membranes13020182

**Published:** 2023-02-02

**Authors:** Shing Fung Lam, Xiaojiao Shang, Raja Ghosh

**Affiliations:** Department of Chemical Engineering, McMaster University, 1280 Main Street West, Hamilton, ON L8S 4L7, Canada

**Keywords:** PEGylated protein, purification, hybrid method, microfiltration, membrane separation

## Abstract

PEGylated proteins are usually purified using chromatographic methods, which are limited in terms of both speed and scalability. In this paper, we describe a microfiltration membrane-based hybrid method for purifying PEGylated proteins. Polyethylene glycol (or PEG) is a lower critical solution temperature polymer which undergoes phase transition in the presence of a lyotropic salt and forms micelle-like structures which are several microns in size. In the proposed hybrid method, the PEGylated proteins are first converted to their micellar form by the addition of a lyotropic salt (1.65 M ammonium sulfate). While the micelles are retained using a microfiltration membrane, soluble impurities such as the unmodified protein are washed out through the membrane. The PEGylated proteins thus retained by the membrane are recovered by solubilizing them by removing the lyotropic salt. Further, by precisely controlling the salt removal, the different PEGylated forms of the protein, i.e., mono-PEGylated and di-PEGylated forms, are fractionated from each other. Hybrid separation using two different types of microfiltration membrane devices, i.e., a stirred cell and a tangential flow filtration device, are examined in this paper. The membrane-based hybrid method for purifying PEGylated proteins is both fast and scalable.

## 1. Introduction

PEGylation, which refers to the covalent attachment of polyethylene glycol (PEG), is one of the most common and proven methods by which physical properties and therapeutic effectiveness of protein biopharmaceuticals could be enhanced [1,2]. The improvements resulting from protein PEGylation include an increase in biological half-life due to increase in size, a decrease in proteolytic degradation and immunogenicity due to shielding, and a decrease in hydrophobicity and the tendency to aggregate due to the presence of the hydrophilic PEG chains [3,4]. Numerous synthetic methods are available for carrying out protein PEGylation [5,6]. After the chemical reaction, the PEGylated protein is purified from unreacted protein and other species present in the reaction mixture, typically using chromatographic methods [7,8,9,10,11,12,13,14,15].

A PEGylated protein can be purified by size exclusion chromatography (based on the fact that it is bigger than its impurities) [7,8], by ion-exchange chromatography (based on the shielding of charged groups present on the protein molecule by the neutral PEG component) [9,10,11,12,13], by hydrophobic interaction chromatography (based on the phase transition of PEG in the presence of salt which make the PEGylated protein apparently more hydrophobic than the unmodified protein) [14,15], and by heparin affinity chromatography [16,17]. The purification of PEGylated proteins using monolith-based chromatography has also been reported [17]. While very pure products can be obtained using these chromatographic methods, the productivity in chromatography is restricted by binding capacity and scalability limitations [18,19,20,21,22,23,24,25]. The large-scale production capability for PEGylated proteins could be significantly enhanced through the use of a high-throughput, non-chromatographic purification step followed by a high-resolution chromatographic step. However, there has been considerably less work done on purification of PEGylated proteins using non-chromatographic methods. Ultrafiltration (UF), which is a permeability-based membrane separation technique, has been proposed as a scalable and high-throughput method for purification of PEGylated proteins [19,20,21,22]. Following a conventional line of thinking, one would expect such a separation to be based on size difference. However, this is not the case, as PEG, unlike a protein, is a relatively flexible molecule which can elongate during ultrafiltration, making size-based separation extremely difficult. Most of the work on the purification of PEGylated proteins using UF is based on the exploitation of electrostatic charge shielding by PEG [19,20,21,22]. Aqueous two-phase systems have also been successfully used for purifying PEGylated proteins [23,24,25]. This technique relies on addition of salt such as citrate which serve both as reaction stopper (thereby avoiding the use of hydroxylamine) as well as phase separator [23]. This approach of combining reaction and separation is referred to as process integration, and this can be done in many different ways [24,25,26,27,28].

PEG is an LCST (lower critical solution temperature) polymer, i.e., it undergoes phase transition from a hydrophilic state to a mildly hydrophobic one when temperature is increased [29]. This phase transition can be made to occur at ambient temperature by the addition of lyotropic salts such as sodium chloride and ammonium sulfate. Upon phase transition, the normally hydrophilic PEG collapses into a relatively hydrophobic entity, and if the salt concentration is increased further, these hydrophobic entities eventually aggregate and form micelle-like structures [30]. It is anticipated that the micellar PEGylated proteins that consist of collapsed PEG chains on the interior and the relatively more hydrophilic protein (at that solution condition) on the exterior would be large enough to be retained by appropriate microporous membranes. Our preliminary studies showed that a majority of these micelles were several microns in size. Therefore, they could potentially be retained by a microfiltration membrane while most of the impurities such as unmodified proteins could be washed out through the membrane. Based on this, a membrane-based hybrid technique for purifying PEGylated proteins could be developed. Membrane-based hybrid separation techniques are those that typically combine a solute insolubilization step with a membrane filtration step [31,32,33,34]. However, unlike protein-precipitation-based hybrid separation [31,32,33,34], the current work is based on the phase transition and micelle formation of the PEG component of PEGylated proteins.

**Figure 1:**
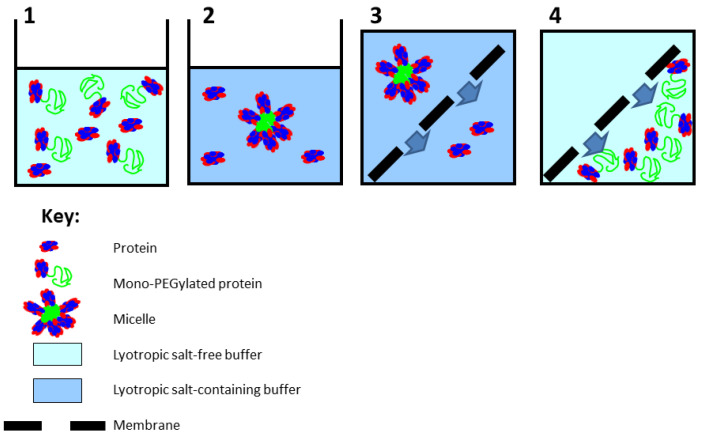
Steps involved in hybrid purification of PEGylated protein.

The working principle of the hybrid purification method proposed in this paper is shown in Figure 1. Step 1 shows the feed material, consisting of a mixture of the unmodified protein and the PEGylated protein; step 2 shows the formation of micron-sized micelle-like structures due to addition of salt; step 3 shows the separation of the unmodified protein and PEGylated protein micelles by microfiltration; and step 4 shows the recovery of the PEGylated protein from the membrane module by reduction in salt concentration, following which the solubilized PEGylated proteins freely pass through the pores of the membrane. The technique was evaluated by examining its applicability for purifying mono-PEGylated human serum albumin (HSA). Hybrid purification was carried out at two different scales of operation: (a) at a small scale using a stirred cell filtration device and (b) at a significantly larger scale using a tangential flow filtration system. The results obtained are discussed.

## 2. Materials and Methods

### 2.1. Materials

Human serum albumin (A8763), FITC-BSA (A9771), sodium cyanoborohydride (156159), sodium acetate, sodium phosphate monobasic (S0751), sodium phosphate dibasic (S0876), ammonium sulfate (A4418), glycine (G8898), barium chloride (202738), hydrochloric acid (258148), 25% glutaraldehyde (G6257), iodine (326143), glycine (G8898), Trizma (T1503), 30% acrylamide (A3699), N,N,N′,N′-Tetramethyethylene diamine (T9281), 70% perchloric acid (77227), and ammonium persulfate (A3678) were purchased from Sigma Aldrich, St. Louis, MO, USA. Sodium dodecyl sulfate (17-1313) was purchased from GE Healthcare, Mississauga, ON, Canada. High quality deionized water (18.2 MΩ cm) was obtained from a DiamondTM NANOpure water purification unit (Barnstead, Dubuque, IA, USA). Isopore membrane (0.22 µm pore size, GTTP04700), PES membrane (5µm pore size, SMWP04700), and PES membrane (0.2 µm pore size, GSWP14250) were purchased from Millipore, Billerica, MA, USA. mPEG-propionaldehyde 10kDa (P1PAL-10) was purchased from Sun Bio Inc., Gyeonggi-do, Republic of Korea.

### 2.2. Protein PEGylation

HSA PEGylation was carried out at room temperature in small flasks with continuous stirring. The reaction mixture consisted of 1 mg/mL HSA, P1PAL-10 (P1PAL-10: HSA molar ratio being 4:1) and 10 mM sodium cyanoborohydride in 100 mM sodium acetate buffer (pH 5.0) as reaction medium. The PEGylation reaction was carried out for 20 h followed by quenching by addition of 1.0 M glycine solution to a final glycine concentration of 10 mM. A batch of FITC-BSA was also PEGylated and quenched using the above protocol. The PEGylated FITC-BSA micelles formed by addition of lyotropic salt were observed using fluorescence microscopy.

### 2.3. Stirred Cell Filtration

**Figure 2:**
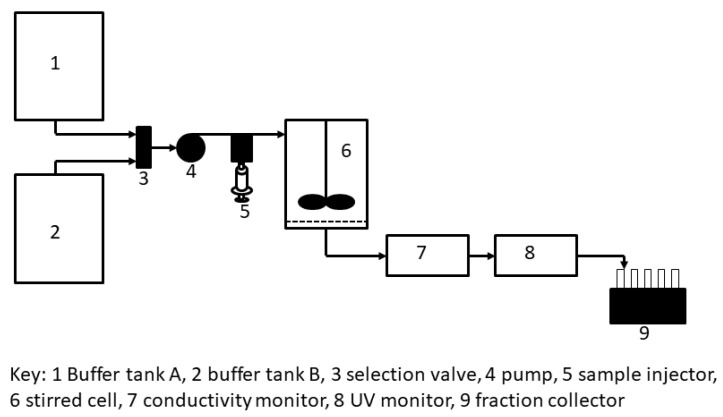
Set-up used for stirred cell membrane filtration-based hybrid separation of PEGylated protein.

The stirred cell filtration set-up in this study is shown in Figure 2. A custom-designed stirred cell filtration module having an effective volume of 13 mL was integrated with an AKTA Prime liquid chromatography system (GE Healthcare). The module was fitted with a stack of 4 membrane discs, each having 28 mm diameter. The top layer was an Isopore membrane with 0.22 µm pores size (Millipore, GTTP04700). This membrane served as the retaining membrane for the PEGylated protein micelles. The remaining layers consisted of regenerated PES membrane discs (Millipore, SMWP04700) with 5 µm pore size. These 3 layers of backing membrane were added to provide mechanical support to the Isopore membranes. The effective salt concentration within the membrane module was adjusted with the AKTA system using a combination of two buffers (buffer A: 1.85 M ammonium sulfate prepared in 100 mM sodium acetate buffer, pH 5.0; buffer B: 100 mM sodium acetate buffer, pH 5.0). The separation experiment was started by pumping a combination of buffer A and buffer B through the membrane module at 3 mL/min flow rate such that the effective ammonium sulfate concentration was 1.65 M. The PEGylation reaction mixture samples (5 mL volume), adjusted to 1.65 M ammonium sulfate, were then injected into the membrane module. In these experiments, the UV absorbance (at 280 nm), conductivity, and pH values were continuously monitored and recorded using Prime View 5.31 (GE Healthcare Bio-Science, Mississauga, ON, Canada). The buffers used for elution were 1.5 M, 1.35 M, and 0 M of ammonium sulfate solutions, in that order. The samples corresponding to the different flow-through and eluted peaks were collected and analyzed by using sodium dodecyl sulfate polyacrylamide gel electrophoresis (SDS-PAGE) and size-exclusion chromatography (SEC).

### 2.4. Tangential Flow Filtration

**Figure 3:**
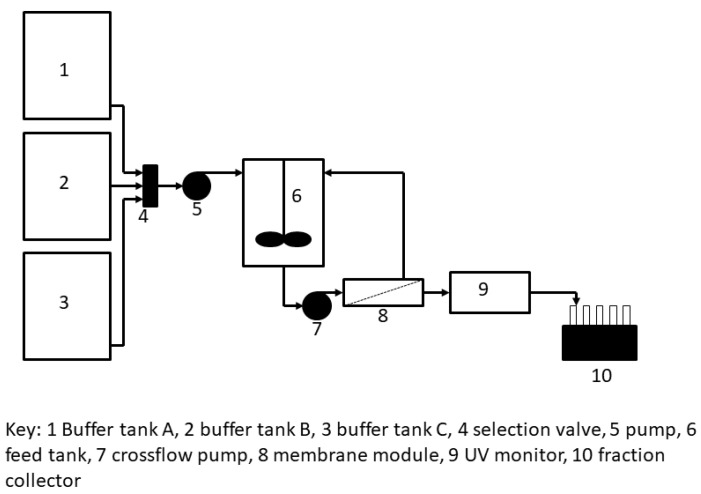
Set-up used for tangential flow-filtration-based hybrid separation of PEGylated protein.

The tangential flow filtration (TFF) set-up used for hybrid separation is shown in Figure 3. Tank 1 contained the PEGylated protein solution; tank 2 contained 1.85 M ammonium sulfate solution prepared in 100 mM sodium acetate buffer, pH 5.0; and tank 3 contained 100 mM sodium acetate buffer, pH 5.0 (i.e., 0 M ammonium sulfate). The custom-designed TFF module housed within it a rectangular piece of PES membrane (GSWP14250, 0.22 µm pores size) having an area of 10 cm^2^. The feed channel depth was 1 mm. As with the stirred cell membrane filtration experiments described in the previous paragraph, the PEGylated protein feed solution was adjusted 1.65 M ammonium sulfate concentration by appropriately proportioning the liquids from tank 1 and tank 2 using an MCP pump. This modified feed solution was pumped to an intermediate feed tank (17 mL volume), from where it was pumped to the tangential flow filtration module at a flow rate of 2 mL/min using a peristaltic pump. Permeate was collected by suction using an P90 pump (GE Healthcare, Mississauga, ON, Canada) at a flow rate of 1 mL/min and this was directed to a UV detector. The retentate from the tangential flow filtration module was sent back to the intermediate feed tank. The buffers used for elution were 1.5 M and 0 M of ammonium sulfate solutions, in that order. The samples corresponded to the different flow-through, and eluted peaks were collected and analyzed by using sodium dodecyl sulfate polyacrylamide gel electrophoresis (SDS-PAGE) and size-exclusion chromatography (SEC).

### 2.5. SEC Analysis

SEC analysis was carried out at a flow rate of 0.5 mL/min using a Superdex 200 10/300 GL column (GE Healthcare Bio-Sciences, Mississauga, ON, Canada) fitted to a Varian HPLC system (Varian, Palo Alto, CA, USA) using 20 mM sodium phosphate buffer of pH 7.0 as mobile phase.

### 2.6. SDS-PAGE

SDS-PAGE experiments were carried out according to the work of Laemmli [35]; 10% non-reducing gels were run using a Hoefer MiniVE system (GE Healthcare Bio-Sciences, Canada). The gels were stained with Coomassie brilliant blue dye to visualize the protein bands.

### 2.7. Microscopy

PEGylated FITC-BSA samples were examined by light and florescence microscopy at a magnification factor of 50×.

## 3. Results and Discussion

**Figure 4:**
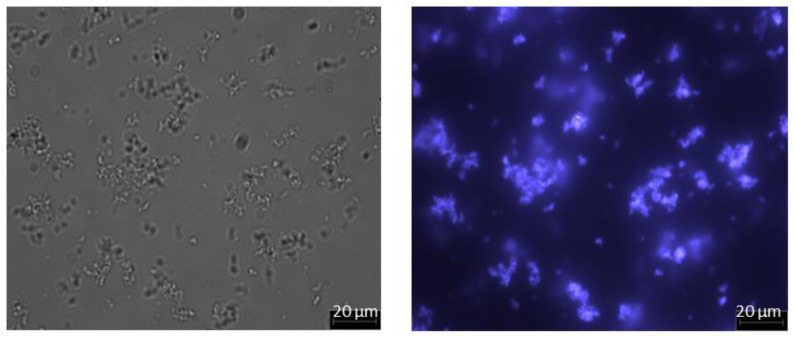
Micrographs of PEGylated FITC-BSA solution adjusted to 1.65 M ammonium sulfate concentration (**left**: image obtained by optical microscopy; **right**: image of same field obtained by fluorescence microscopy; magnification: 50×).

When sufficient salt is added, the PEG component of the PEGylated protein undergoes phase transition, thereby becoming more hydrophobic and forming a micellar structure [29,30]. Figure 4 shows the optical and fluorescent micrographs obtained with PEGylated FITC-BSA solution adjusted to 1.65 M ammonium sulfate concentration (prepared as discussed in Section 2.2) at 50× magnification. Both images indicate presence of micellar structures in the size range of 1 to 10µm formed by PEGylated FITC-BSA. Based on this, it could be expected that PEG-HSA would behave in a similar way, i.e., by forming micelles in the micron size range. It could also be anticipated that these micelles would be retained by microfiltration membranes, and thereby these could easily be separated from any unreacted protein.

**Figure 5:**
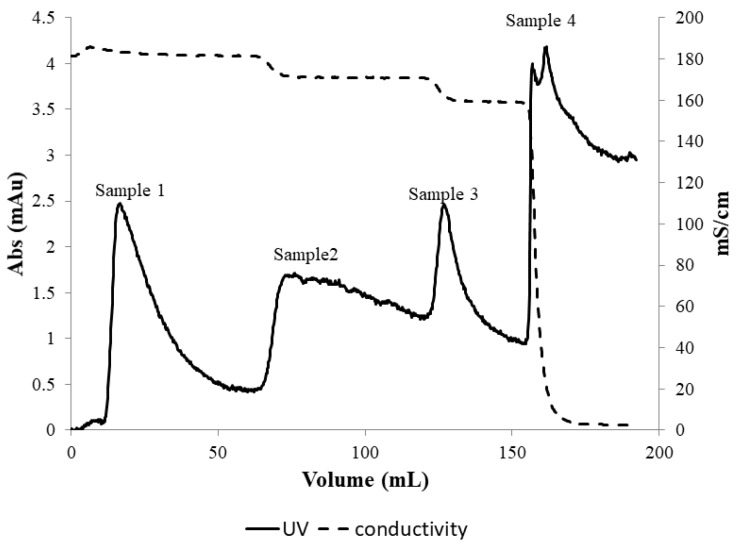
Hybrid separation of PEG-HSA using stirred cell membrane module. Samples 1–4 correspond to the 1.65, 1.5, 1.35, and 0 M ammonium sulfate concentration in the stirred cell membrane module.

Figure 5 shows the UV absorbance and conductivity profiles obtained from the experiment for PEGylated HSA purification using the stirred cell microfiltration protocol described in Section 2.3. The peak labeled sample 1 in the figure represented the flow-through corresponding to 1.65 M ammonium sulfate concentration within the membrane module. Since PEG-HSA was expected to form micron-sized micellar structures at this salt concentration (which were retained by the 0.22-micron membrane housed within the stirred cell module), it could be presumed that the peak was due to unreacted HSA in the feed sample that could pass through the membrane. When the ammonium sulfate concentration was reduced in three steps to 1.5 M, 1.35 M and 0 M respectively, three eluted “peaks” were obtained corresponding to each of these steps. Samples corresponding to each of these peaks were collected and analyzed by SDS-PAGE and SEC.

**Figure 6:**
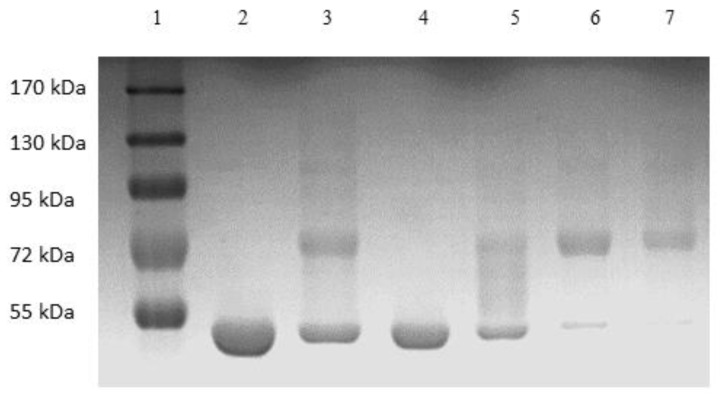
Image of Coomassie-blue-stained SDS-PAGE gel of sample obtained by stirred cell hybrid separation (lane 1: MW markers, lane 2 HSA, lane 3 PEG reaction mixture, lane 4 sample 1 (1.65 M flow-through), lane 5 sample 2 (1.5 M eluate), lane 6 sample 3 (1.35 M eluate), lane 7 sample 4 (0 M eluate)).

**Figure 7:**
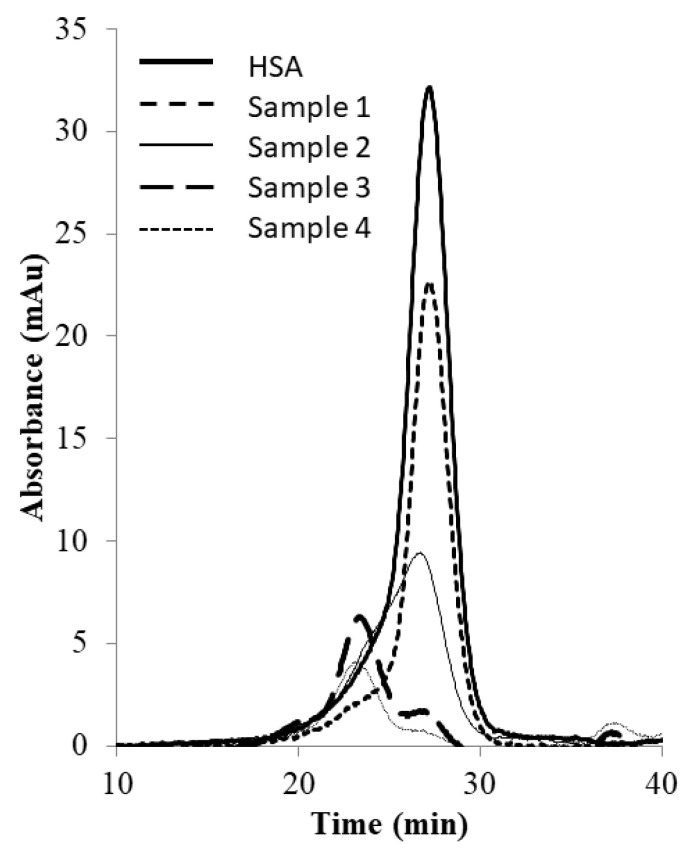
SEC chromatograms of samples obtained by stirred cell membrane filtration-based hybrid separation (sample 1: 1.65 M flow-through, sample 2: 1.5 M eluate, sample 3: 1.35 M eluate, sample 4: 0 M eluate).

Figure 6 shows the image of the Coomassie-blue-stained SDS-PAGE gel corresponding to the “peak” samples collected during the stirred cell microfiltration-based PEG-HSA purification experiment described in the previous paragraph. Samples loaded in lanes 2 and 3 corresponded to pure HSA and unfractionated PEG-HSA reaction mixture respectively. The bands on lane 3 indicate that mono-PEGylated HSA was the main PEGylation product, while smaller amounts of higher PEGylated forms were also synthesized. The single band in lane 4 indicate that Sample 1, i.e., the flow-through obtained corresponding to 1.65 M ammonium sulfate, contained only HSA. Therefore, PEG-HSA was quantitatively retained within the membrane module at this salt concentration. The eluate obtained at 1.5 M ammonium sulfate concentration (Sample 2) contained similar amounts of HSA and mono-PEG-HSA. Samples 3 and 4 were made up almost entirely of PEG-HSA (i.e., were largely or completely free of unreacted HSA), with Sample 3 containing mainly mono-PEG-HSA and Sample 4 containing some of the higher PEGylated forms. Figure 7 shows the SEC chromatograms obtained with samples collected during the stirred cell microfiltration-based PEG-HSA purification experiment. As expected from the SDS-PAGE results shown in Figure 6, single SEC peaks were obtained with pure HSA and Sample 1. Sample 2 consisted of a mixture of HSA and mono-PEGylated HSA as evident from the broad bump instead of a defined single SEC peak. Samples 3 and 4 consisted almost entirely of PEG-HSA (i.e., these samples were quite pure). Thus, the SDS-PAGE results shown in Figure 6 were consistent with the SEC results shown in Figure 7. These experiments also provide preliminary evidence that the stirred cell microfiltration-based hybrid separation technique could indeed be used for separation of PEG-HSA from the PEGylation reaction mixture.

**Figure 8:**
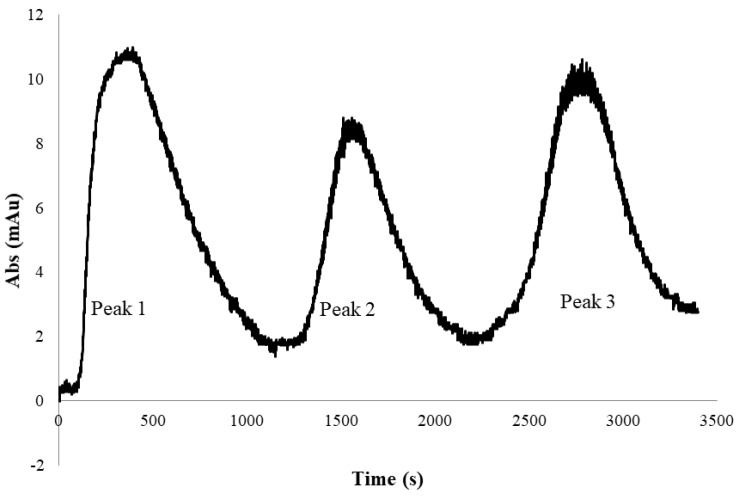
UV absorbance profile of the permeate stream obtained during tangential flow hybrid separation of PEGylated HSA (peak 1: 1.65 M flow-through, peak 2: 1.5 M eluate, peak 3: 0 M eluate).

Figure 8 shows the UV absorbance profile obtained from the experiment conducted for PEGylated HSA purification using the tangential flow membrane filtration system described in Section 2.4. The peak 1 in the figure corresponded to the flow-through obtained at 1.65 M ammonium sulfate concentration. As in the stirred cell microfiltration experiment, PEG-HSA being micron-sized would be expected to be quantitatively retained by the 0.2-micron membrane housed within the TFF module. It could therefore be presumed that peak 1 was due to unreacted HSA present in the feed sample that simply flowed through the membrane. When the ammonium sulfate concentration was reduced in two steps to 1.5 M and 0 M, two eluted “peaks” were obtained corresponding to each of these steps. Samples corresponding to each of these peaks were collected and analyzed by SDS-PAGE and SEC.

**Figure 9:**
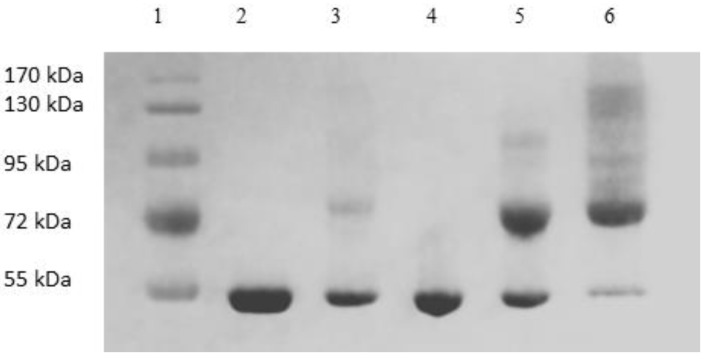
Image of Coomassie-blue-stained SDS-PAGE gel of samples obtained by tangential flow-based hybrid separation (lane 1: MW markers, lane 2: HSA, lane 3: PEG reaction mixture, lane 4: peak 1 (1.65 M flow-through), lane 5: peak 2 (1.5 M eluate), lane 6: peak 3 (0 M eluate)).

**Figure 10:**
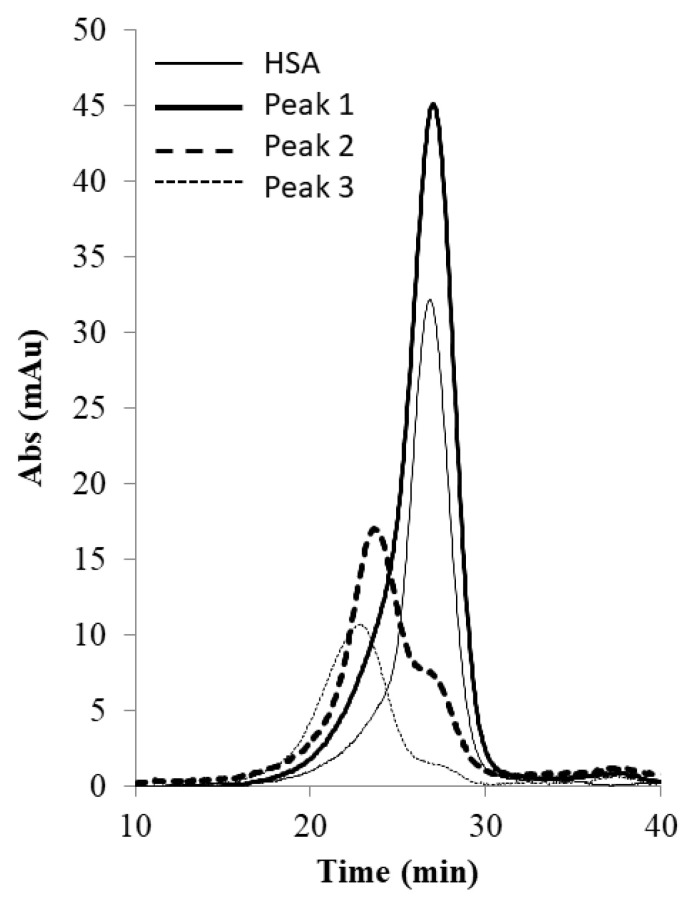
SEC chromatograms of samples obtained by tangential flow-filtration-based hybrid separation (peak 1: 1.65 M flow-through, peak 2: 1.5 M eluate, peak 3: 0 M eluate).

Figure 9 shows the image of the Coomassie-blue-stained SDS-PAGE gel obtained with the samples collected during the tangential flow-filtration-based PEG-HSA purification experiment described in the previous paragraph. Samples loaded in lanes 2 and 3 were pure HSA and unfractionated PEG-HSA reaction mixture respectively. Peak 1, i.e., the flow-through obtained corresponding to 1.65 M ammonium sulfate contained only HSA (lane 4). The eluate obtained at 1.5 M ammonium sulfate concentration (lane 5) contained both HSA and mono-PEG-HSA. The eluate sample obtained at 0 M ammonium sulfate (lane 6) was made up almost entirely of PEG-HSA including both mono-PEG-HSA and higher PEGylated forms. Figure 10 shows the SEC chromatograms obtained with samples collected during the tangential flow-filtration-based PEG-HSA purification experiment. As expected from the SDS-PAGE results shown in Figure 9, single SEC peaks were obtained with pure HSA and the flow-through obtained at 1.65 M ammonium sulfate. The eluate obtained at 1.5 M ammonium sulfate consisted of mostly mono-PEGylated HSA and smaller amounts of HSA as evident from the composite peak skewed towards mono-PEGylated HSA. The eluate obtained at 0 M ammonium sulfate consisted almost entirely of PEG-HSA (i.e., the sample was quite pure). Once again, the SDS-PAGE results shown in Figure 9 are consistent with the SEC results shown in Figure 10. These experiments provide further evidence that the microfiltration-based hybrid separation technique could indeed be used for separation of PEG-HSA from the PEGylation reaction mixture. Based on these results, a high recovery of PEG-HSA could be expected. The results obtained with the stirred cell microfiltration experiments were also very consistent with those obtained with the tangential flow filtration experiments, indicating that the method was scalable. However, these results are preliminary in nature and the work presented in this paper is meant to be a proof-of-concept study for the proposed hybrid separation of PEGylated proteins. The results discussed in the paper clearly indicate that the separation performance could be improved through proper process optimization. This would involve optimization of the feed condition, filtration parameters, proper selection of membranes, and optimization of elution conditions. While the current study does indicate the hybrid techniques would be suitable for purification of PEGylated proteins, it does not demonstrate superiority over chromatography. This can only be achieved by performing an objective head-to-head comparison with an equivalent chromatographic separation process. This will be included as part of a follow-up study. The follow up study will also include quantitative aspects such as mass balance, purity analysis, and determination of product recovery. In hybrid membrane processes, proper membrane selection is of critical importance. The membrane used should be able to quantitatively retain the precipitated/micellar species while allowing unhindered passage of soluble species. Buffer consumption is an important factor in a bioseparation process, and this factor could influence the type of equipment used for the hybrid separation process. With a stirred cell device, the buffer consumption would be quite high. However, much lower buffer consumption could be expected when using a tangential flow filtration system. The buffer consumption in a stirred cell could be significantly decreased by stopping stirring during the elution phase and thereby allowing the eluate to be obtained as a more concentrated fraction. The use of this strategy has been discussed in the context of immunoglobulin G purification using precipitation-based hybrid separation [33].

## 4. Conclusions

The results discussed in the paper provide proof of concept for the proposed hybrid purification of PEGylated protein from PEGylation reaction mixture. The addition of the lyotropic salt resulted in the formation of micron sized micelles of the PEGylated protein. These micelles could be retained by using appropriate microfiltration membrane which simultaneously allowed the unreacted protein to flow through. The retained PEGylated protein could be recovered by elution from the membrane device by lowering the lyotropic salt concentration. The results obtained with the stirred cell microfiltration experiments were consistent with those obtained with the tangential flow filtration device, indicating that the method was scalable. The separation performance could be improved through proper process optimization, which would involve optimization of feed condition, filtration parameters, proper selection of membrane and optimization of elution conditions. The optimized methods thus developed could potentially overcome some of the processing limitations typically associated with resin-based chromatography.

## Data Availability

Data will be made available upon reasonable request.
